# Impacts of Fertilization Optimization on Soil Nitrogen Cycling and Wheat Nitrogen Utilization Under Water-Saving Irrigation

**DOI:** 10.3389/fpls.2022.878424

**Published:** 2022-05-19

**Authors:** Zhen Zhang, Zhenwen Yu, Yongli Zhang, Yu Shi

**Affiliations:** Key Laboratory of Crop Ecophysiology and Farming System, Ministry of Agriculture, Shandong Agricultural University, Taian, China

**Keywords:** wheat, nitrification intensity, denitrification intensity, split nitrogen fertilization, ammonia volatilization

## Abstract

Scholars have proposed the practice of split nitrogen fertilizer application (SNFA), which has proven to be an effective approach for enhancing nitrogen use efficiency. However, the combined effects of SNFA on wheat plant nitrogen use efficiency, ammonia (NH_3_) emission flux, as well as the rates of nitrification and denitrification in different ecosystems remain unclear. Meanwhile, few studies have sought to understand the effects of the split nitrogen fertilizer method under water-saving irrigation technology conditions on nitrogen loss. The current study assessed soil NH_3_ volatilization, nitrification, and denitrification intensities, as well as the abundance of nitrogen cycle-related functional genes following application of different treatments. Specifically, we applied a nitrogen rate of 240 kg⋅ha^–1^, and the following fertilizer ratios of the percent base to that of topdressing under water-saving irrigation: N1 (basal/dressing, 100/0%), N2 (basal/dressing, 70/30%), N3 (basal/dressing, 50/50%), N4 (basal/dressing, 30/70%), and N5 (basal/dressing, 0/100%). N3 treatment significantly reduced NH_3_ volatilization, nitrification, and denitrification intensities, primarily owing to the reduced reaction substrate concentration (NO_3_^–^ and NH_4_^+^) and abundance of functional genes involved in the nitrogen cycle (*amoA-*AOB, *nirK*, and *nirS*) within the wheat-land soil. ^15^N tracer studies further demonstrated that N3 treatments significantly increased the grain nitrogen accumulation by 9.50–28.27% compared with that under other treatments. This increase was primarily due to an increase in the amount of nitrogen absorbed by wheat from soil and fertilizers, which was caused by an enhancement in total nitrogen uptake (7.2–21.81%). Overall, N3 treatment (basal/dressing, 50/50%) was found to effectively reduce nitrogen loss through NH_3_ volatilization, nitrification and denitrification while improving nitrogen uptake by wheat. Thus, its application will serve to further maximize the yield and provide a fertilization practice that will facilitate cleaner wheat production in the North China Plain.

## Introduction

The most important crop used to model cultivation on the North China Plain (NCP) is winter wheat. In an attempt to obtain high grain yields, excessive amounts of nitrogen fertilizer are often used. In fact, the rate applied annually can be as high as 325 kg N ha^–1^, which poses a significant problem as nitrogen fertilizers represent the major source of reactive nitrogen (Sun et al., 2020). Although this nutrient is indispensable for wheat ecosystems, an inadvertent effect can include high nitrogen loss and low fertilizer efficiency. In fact, the nitrogen use efficiency in wheat crops commonly ranges from 30 to 40% following fertilization (Wu et al., 2019). However, the excessive use of nitrogen fertilizers contributes to rapid losses of nitrogen through volatilization in the form of ammonia (NH_3_), nitrification, denitrification, and surface runoff (Peng et al., 2006). Therefore, improvements in wheat nitrogen use efficiency and reduction of nitrogen loss are essential for the sustainability of agricultural ecosystems.

Soil denitrification and nitrification are vital processes in the global nitrogen cycle (Harter et al., 2014), as well as in the generation of environmental pollution, and plant metabolism (Li M. N. et al., 2018). Previous researches have shown that high rates of nitrification and denitrification can result in reduced nitrogen use efficiency by crops and a corresponding increase in loss of nitrogen to the atmosphere (e.g., as nitrogen oxides) or leaching into surface or ground waters (Yang et al., 2017). Ammonium (NH_4_^+^) is oxidized to nitrate (NO_3_^–^) during nitrification, with nitrite serving as an intermediate. These processes are catalyzed separately by nitrite oxidoreductase and ammonia monooxygenase, respectively. Meanwhile, denitrification is a sequential reduction by which NO_3_^–^ is reduced to N_2_O and/or dinitrogen gas (N_2_) *via* NO_2_^–^ and nitric oxide (NO), which are catalyzed by a series of enzymes that include copper-containing nitrite reductase and cytochrome cd1-containing nitrite reductase. In addition, studies have shown that nitrogen fertilization affects these processes by affecting the soil microbial characteristics (Elrys et al., 2021). Meanwhile, some functional genes have been used as molecular markers to investigate the dynamics of microbial communities responsible for specific nitrogen transformation processes in various environments, including field soils (Petersen et al., 2012). The most frequently studied nitrogen cycling marker genes include *amoA* (encoding ammonia monooxygenase), *nirK* (encoding Cu nitrite reductase), and *nirS* (encoding cd1 nitrite reductase). Quantifying the abundance of nitrogen cycling functional genes can provide a good estimate of the effects of nitrogen fertilization on soil nitrogen transformation. For instance, a previous study investigated the effect of nitrogen fertilization on soil nitrogen cycling gene abundances and found that *amoA*-AOB were much more responsive than *amoA*-AOA to nitrogen fertilization. Specifically, the effect size of nitrogen fertilization on *amoA*-AOB was 9 times higher than that of *amoA*-AOA (Yang X. D. et al., 2020). Similar to *amoA*-AOA and *amoA*-AOB, genes involved in denitrification are also significantly upregulated by nitrogen fertilization (Antonia et al., 2020). Thus, understanding the effects of soil functional genes of specific nitrogen transformation processes on soil nitrification and denitrification is vital to promote the efficient utilization of nitrogen in agriculture through the application of various strategies, including amendment of soils and rational fertilization.

Soil nitrogen loss can occur *via* NH_3_ volatilization, soil nitrification and denitrification processes, plant uptake, runoff, and leaching (Deng et al., 2015). Among these pathways, plant nitrogen uptake can prove beneficial by increasing grain yield (Waldrop et al., 2017). However, NH_3_ volatilization often dominates the other pathways, particularly in dryland soils (Abdo et al., 2021). The proportion of total nitrogen lost as NH_3_ from nitrogen fertilizers varies from 9 to 40% (Fu et al., 2020). Meanwhile, the primary air pollutants now include atmospheric NH_3_, which is rapidly deposited on the Earth’s surface within 4–5 km of its sources (Behera et al., 2013). It is, therefore, crucial to manage nitrogen fertilizers in a manner that minimizes their effects on the environment. Moreover, NH_3_ volatilization is also impacted by soil moisture. As such, our previous research proposed a water-saving irrigation technology (WCT) based on measuring soil moisture at the key stage of wheat growth (Man et al., 2014). Compared with traditional quantitative irrigation, this technology not only reduces water use, but also improves wheat growth and yield. However, few studies have sought to understand the effects of the split nitrogen fertilizer method under WCT conditions on nitrogen loss.

The main processes associated with soil nitrogen conversion, such as nitrification and denitrification, significantly impact soil nitrogen loss, as well as the wheat nitrogen use efficiency. Meanwhile, previous studies have indicated that when nitrogen fertilization rates are high, the NH_4_^+^ concentration in the soil and its volatilization as NH_3_ increase (Friedel and Gabel, 2001). Therefore, factors such as NH_3_ emissions must also be considered when evaluating the impacts of nitrogen fertilization on wheat nitrogen use efficiency and nitrogen loss (Huang et al., 2013). Although previous researches have examined the individual effects of nitrogen fertilizer management on wheat plant nitrogen use efficiency, NH_3_ emission flux, as well as the rates of nitrification and denitrification in different ecosystems, few experiments have investigated their combined effects. In this study, we combine q-PCR and ^15^N stable isotope tracing to explore the potential effects of split nitrogen fertilizers on NH_3_ emission flux and the rates of denitrification and nitrification, as well as the utilization to plant nitrogen. Accordingly, the study aims were to (1) examine the effect of split nitrogen fertilizer application on the soil NH_3_ volatilization, denitrification, nitrification, and the genes abundance encoding key enzymes involved in soil nitrogen cycling; and (2) investigate the effects of split nitrogen fertilizer application on nitrogen use efficiency in typical winter wheat systems. This study was conductive to a better understanding of the nitrogen management measures that decreased nitrogen loss reduction mechanism, provide inspiration for the regulation of nitrogen-increased nitrogen use efficiency improve and was expected to calibrate the greenhouse gas emission reduction mechanism potential of nitrogen management measures in soils.

## Materials and Methods

### Experimental Site Description

The field experiments took place in Xiaomeng town, Jining City, Shandong Province, China, during the growing seasons of winter wheat from 2016 to 2017 and 2017 to 2018 (Supplementary Figure 1). This site has a typical warm continental climate. The average annual climate factors include an average annual temperature of 15°C, average annual precipitation of 600 mm. Summer maize-winter wheat is the major crop rotation regime in this region. According to the FAO classification, the wheat-land soil is loam. The properties were as follows in the layer of soil tillage (0–20 cm): concentration of organic matter, 14.20 g⋅kg^–1^; total available phosphorus, 38.11 mg⋅kg^–1^; total available potassium, 129.44 mg⋅kg^–1^; available nitrogen total N, 122.60 mg⋅kg^–1^; pH, 7.6 and total nitrogen, 1.13 g⋅kg^–1^. The data for the mean monthly precipitation are shown in Supplementary Figure 2.

### Split Nitrogen Fertilizer Treatment and Water-Saving Irrigation

The winter wheat cultivar of “Jimai 22” was used in this study. In this study, we assessed the most widely used winter wheat variety in the surrounding area (cv. Jimai 22). It was the first variety planted in China for 9 years in a row, with a cumulative planting area of 270 million mu. The wheat was seeded on October 12th, October 24th and harvested on June 8th, June 7th in 2016–2017 and 2017–2018, respectively. The density was 1.8 million⋅ha^–1^.

The field experiment consisted of five different split nitrogen fertilizer applications at an application rate of 240 kg⋅ha^–1^ (basal/dressing, 100/0%, 70/30%, 50/50%, 30/70%, 0/100%; hereafter referred to as N1, N2, N3, N4, and N5, respectively) with a randomized plot design (Table 1). Each application was performed in triplicate, resulting in a total of 15 plots (plot area 20 m^2^). The selected rate of fertilizer application is commonly used by the local farmers. Single superphosphate (P_2_O_5_ 12%) and potassium chloride (K_2_O 60%) were applied to provide P (P_2_O_5_ 150 kg⋅ha^–1^) and K (112.5 K_2_O kg⋅ha^–1^), respectively. The basal fertilizer was comprised of P and K, while the nitrogen was applied in two split applications. All potash and phosphate fertilizers, as well as the basal nitrogen fertilizer were spread over the soil surface before the wheat was sown. A rotary cultivar was used to immediately mix the soil to a depth of 20 cm. During the jointing stage, nitrogen fertilizer was applied to create furrows that were immediately covered.

**TABLE 1:** Agronomic managements under different split nitrogen fertilizer treatment.

Treatment	Fertilizer regime			Irrigation regime	
	Seedling stage		Jointing stage	Jointing stage	Anthesis stage
N1	240 N kg⋅ha^–1^ as urea	150 P_2_O_5_ kg⋅ha^–1^ as superphosphate and 112.5 K_2_O kg⋅ha^–1^ as potassium chloride	0 N kg⋅ha^–1^ as urea	Relative water content in 0∼40 cm soil layer is supplemented to 70% according to soil moisture content in 0∼40 cm soil layer.	
N2	168 N kg⋅ha^–1^ as urea		72 N kg⋅ha^–1^ as urea		
N3	120 N kg⋅ha^–1^ as urea		120 N kg⋅ha^–1^ as urea		
N4	72 N kg⋅ha^–1^ as urea		168 N kg⋅ha^–1^ as urea		
N5	0 N kg⋅ha^–1^ as urea		240 N kg⋅ha^–1^ as urea		

The soil moisture was measured to manage this parameter based on a WCT. The relative water content in the 0∼40 cm soil layer was supplemented to 70% at the jointing and anthesis. The amounts of irrigation was calculated using the method of Man et al. (2014). All irrigation processes involved the use of a hose, and the amount of water used to irrigated each plot was determined manually and recorded with a water meter. The detailed nitrogen fertilizer application and irrigation regimes are shown in Table 1. The fields were managed according to the local practices of farming with standard applications of herbicides and pesticides.

### NH_3_ Volatilization Measurement

NH_3_ was collected from October 2016 to June 2018 *via* the ventilation method. The sampling stages and times are shown in Supplementary Table 1. A polyvinyl chloride (PVC) cylinder (25 cm height, 15 cm diameter) was inserted 70 mm into the soil. The cylinder had a sponge soaked with phosphoglycerol (5%, V/V, phosphoric acid and 4%, V/V, glycerol) near its top to absorb ambient NH_3_ and one close to the bottom to collect NH_3_ from the soil (Dong et al., 2019). The sponges were collected each day for 1 week after each fertilizer application and at each growth stage. The NH_3_ that the sponges had trapped was extracted with 300 mL of 1 mol⋅L^–1^ KCl. The extracted NH_4_^+^ solution was then analyzed with an AA3 continuous flow analyzer (Bran+Luebbe company, Hamburg, Germany). The NH_3_ flux was calculated using the formula described by Yang G. Y. et al. (2020). The cumulative NH_3_ volatilization was calculated as the integral sum of the gas emissions from the stages that were sampled. The NH_3_ volatilization factor and yield-scaled volatilization rate were calculated using the following formulas (Yang G. Y. et al., 2020):


$${\mathrm{NH}}_{3}\mathrm{volatilizaiton}\mathrm{factor}\left(\%\right)=\left(N_{fertilizer}-N_{control}\right)/\left(F_{n}\right)$$

$$\mathrm{Yield}\text{-}\mathrm{scale}{\mathrm{NH}}_{3}\mathrm{volatilizaiton}\left(\mathrm{kg}Nt^{-1}\mathrm{grain}\right)=N_{fertilizer}/G$$

Where *N*_*fertilizer*_ and *N*_*control*_ are the total cumulative NH_3_ that had become volatilized during the entire wheat growing season under the nitrogen fertilizer treatment and control, respectively. *F*_*n*_ is the total nitrogen that had been applied (240 kg⋅ha^–1^). *G* is the grain yield.

### ^15^N Measurement

We conducted isotopic labeling microzone experiments with ^15^N urea during the wheat growing season in 2017 and 2018. The micrograph (area size of 45 cm × 15 cm, depth, 30 cm) was created using an iron sheet (Supplementary Figure 3) and processed in the same manner as the field map. In A, ^15^N-urea and urea were applied during the base dressing and jointing stages, respectively, and in B, urea and ^15^N-urea were applied during the base dressing and jointing, respectively, using the appropriate rates according to the treatment (Chen et al., 2019). In both wheat growing seasons, the plants were sampled from each microzone during the maturity stage. Each sample was first rinsed with running water and transported to the laboratory where they were dried in an oven at 105°C for 30 min and subsequently at 70°C to a constant weight. In the final step, the sample were ground using a ball mill. Stable isotope tests were performed using an element analyzer (Flash 2000HT, Thermo Fisher Scientific, Waltham, MA, United States) and an isotope mass spectrometer (Delta V advantage, Thermo Fisher Scientific, Massachusetts, USA) to determine the ^15^N atomic percentage of the sample. Since the results of the two study growing seasons are similar, only data for 2017–2018 are included here. All indexes were calculated using the formulas (Chen et al., 2019):


(1) $$\mathrm{NDFF}\left(\%\right)=\left[\left({\mathrm{AT}\%}^{15}N_{1}-0.3663\right)\times 100\right]/\left({\mathrm{AT}\%}^{15}N_{2}-0.3663\right)$$

(2) $$\mathrm{NDF}\left(\mathrm{mg}\cdot {\mathrm{stem}}^{-1}\right)=\mathrm{NDFF}\times \mathrm{nitrogen}\mathrm{content}\mathrm{in}\mathrm{plant}\mathrm{components}$$

(3) NDFS=Nuptake-NDF

(4) NAAG=Grainweight×grainnitrogencontent

(5) TNAA=plantweight×plantnitrogencontent

(6) $$\mathrm{SN}={\mathrm{NO}}_{3}^{-}\mathrm{accumulation}\mathrm{amount}+{\mathrm{NH}}_{4}^{+}\mathrm{accumulation}\mathrm{amount}$$

Where NDFF represents the percentage of nitrogen obtained from nitrogen fertilizer, AT% ^15^N_1_ represents the atomic percentage of ^15^N, and AT% ^15^N_2_ represents the atomic percentage of ^15^N in the fertilizer. Additionally, 0.3663 is the standard value for natural ^15^N abundance and nitrogen absorption represents the total nitrogen in the aboveground biomass. NDFF and NDF calculated microplots A (base fertilizer application ^15^N) and B (^15^N applied at jointing), respectively.

### Soil Sample Collection and Preparation

The soil in the 0∼10 cm layer was sampled from each treatment isotopic labeling microzone plot at the stages of wheat jointing, anthesis and maturity (Meng et al., 2020). Each soil sample was a mixture of five randomly selected locations in a given plot. The samples were then passed through a 1 mm sieve and divided into three fractions, mixed thoroughly, and stored at 4°C for subsequent analysis of inorganic nitrogen content (NH_4_^+^ and NO_3_^–^). An aliquot was air dried and ^15^N abundance was determined through a 1-mm sieve. The final sample was freeze-dried and stored at −80°C for subsequent DNA extraction and real-time PCR analysis.

### Total Soil DNA Extraction, q-PCR, and Cloning of Bacterial Genes

Soil DNA was extracted from each samples using a FastDNA Spin Kit for Soil (MP Biomedicals, LLC., Solon, OH, United States) according to the manufacturer’s instructions. The DNA was then stored at −80°C and analyzed within 3 days. A Nanodrop^®^ND-2000 UV-vis spectrophotometer (NanoDrop Technologies, Wilmington, DE, United States) was used to quantify the DNA and determine its purity. To quantify the abundance of *amoA-*AOA, *amoA-*AOB, *nirS*, and *nirK*, quantitative polymerase chain reaction (q-PCR) assays were performed in triplicate using a LightCycler 480 (Roche Applied Science, Basel, Switzerland). The PCR conditions and primers are presented in Supplementary Table 2. The standard curves were prepared as previously described (Li et al., 2017). A plasmid that contained 10^2^–10^9^ copies μL^–1^ was obtained by serial 10 × dilutions. A q-PCR assay was then performed in triplicate to provide an external standard curve to quantify gene copies. The amplification efficiency of target genes ranged from 92.3 to 105.2% and the *R* values were between 0.996 and 0.999. All results analyzed using the StepOnePlus™ Real-Time software (Applied Biosystems, Foster City, CA, United States). The data were transformed into the number of copies of DNA per gram dry soil.

### Nitrification Intensity and Denitrification Intensity, Soil NO_3_^–^ and NH_4_^+^ Content

We conducted an *in situ* experiment on nitrogen mineralization during the growing season of winter wheat in 2017 and 2018. Since most mineralization takes place in the top 2.5–5 cm of soil (Norton et al., 2004; Schimel et al., 2007), we focused on the nitrification intensity and denitrification intensity in the top 10 cm soil layer.

The nitrification intensity were measured using the shaking mud method, as previously described (Yao et al., 2011), whereas the denitrification intensity was measured *via* the acetylene suppression technique (Liu et al., 2014). The specific operation steps were determined according to Meng’s method (Meng et al., 2020).

The concentrations of NH_4_^+^ and NO_3_^–^ were measured using a continuous flow analytical system (Futura Continuous Flow Analytical System, Alliance Instruments, Eragny-Sur-Oise, France) after the samples had been extracted with 1 mol⋅L^–1^ KCl.

### Statistical Analyses

All data were analyzed using two-way analysis of variance (ANOVA) in the SPSS 18.0 (IBM Corp., Armonk, NY, United States) statistical software package to test whether significant differences existed between the split nitrogen treatments in both growth seasons.

## Results

### Effect of Split Nitrogen Fertilizer Application on NH_3_ Volatilization

**FIGURE 1:**
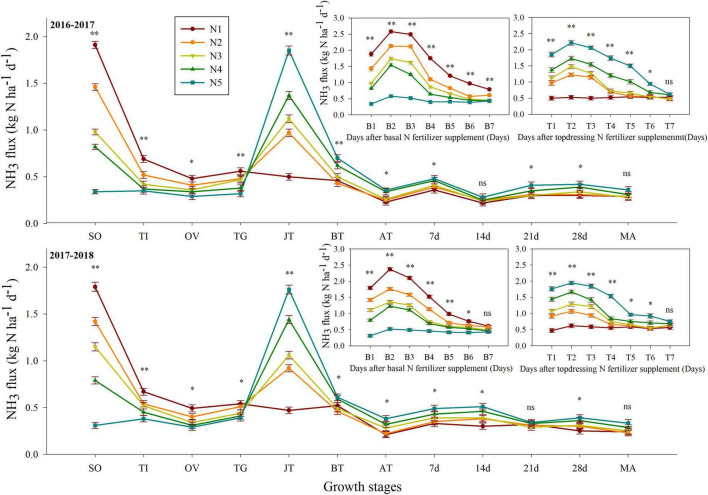
Effect of split nitrogen fertilizer on NH_3_ flux. B1, B2, B3, B4, B5, B6, B7 is the 1, 2, 3, 4, 5, 6, 7 days after basal nitrogen fertilizer supplement; and T1, T2, T3, T4, T5, T6, T7 is the 1, 2, 3, 4, 5, 6, 7 days after topdressing nitrogen fertilizer supplement. *, ^**^ significant at the 0.05 and 0.01 probability levels, respectively, ns, no significant. N1 (basal/dressing, 100/0%), N2 (basal/dressing, 70/30%), N3 (basal/dressing, 50/50%), N4 (basal/dressing, 30/70%), and N5 (basal/dressing, 0/100%).

In general, the peak daily NH_3_ was detected 2 days after each application of nitrogen fertilizer and then decreased to relatively low levels 6–7 days after each nitrogen fertilizer application (Figure 1). Table 2 presents the cumulative volatilization of NH_3_ under different treatments with split nitrogen during the 2017 and 2018 seasons of wheat growing. Specifically, the cumulative NH_3_ volatilization across all nitrogen treatments varied between 16.65–19.23 kg N ha^–1^ d^–1^ and 15.97–17.91 kg N ha^–1^ d^–1^ in both growing seasons. The percent cumulative volatilization of NH_3_ under the N3 treatment was 5.56–13.42% and 5.22–10.83%, lower than those under N1 or N2 and N4 or N5 treatments, respectively. Moreover, the largest decreases in the volatilization factor for NH_3_ and yield-scaled volatilization of NH_3_ were observed under the N3 treatment. N3 treatment decreased the volatilization factor for NH_3_ by 9.43–21.36% and the yield-scaled volatilization by 9.20–18.16% in both growing seasons compared with that under the N1 or N2 and N4 or N5 treatments, respectively. Similarly, under the N3 treatment, the yield-scaled NH_3_ volatilization decreased by 10.76–22.27%, 9.91–22.78% in both growing seasons, respectively. In conclusion, N3 significantly decreased NH_3_ volatilization.

**TABLE 2:** Effect of split nitrogen fertilizer on cumulative NH_3_ volatilization, NH_3_ volatilization factor, and yield-scaled NH_3_ volatilization.

Year	Treatment	Cumulative NH_3_ volatilization (kg N ha^–1^)			NH_3_ volatilization factor (%)	Yield-scaled NH_3_ volatilization (kg N t^–1^ grain)
		BF	TF	Total		
2016–2017	N1	11.7a	3.64e	19.23a	5.01a	2.56a
	N2	8.82b	5.62d	18.08b	4.53b	2.28b
	N3	6.81c	6.26c	16.65c	3.94d	1.99c
	N4	5.68d	8.14b	17.63b	4.35c	2.23b
	N5	3.07e	10.92a	17.95b	4.48b	2.49a
2017–2018	N1	10.13a	3.91e	17.91a	4.46a	2.59a
	N2	7.81b	5.36d	16.87b	4.03b	2.22c
	N3	6.13c	6.14c	15.97c	3.65c	2.00d
	N4	5.38d	7.52b	16.85b	4.02b	2.27c
	N5	3.04e	9.73a	16.88b	4.06b	2.42b
ANOVA (*F*-value)						
Nitrogen treatment		43.284**	63.118**	2.387*	2.428*	88.986**

* and ** indicate significant differences at the 0.05 and 0.01 probability levels, respectively. Values followed by different letters within the same column are significantly different at P < 0.05. BF, The cumulative volatilization of NH_3_ for seven consecutive days after basal nitrogen fertilizer supplement; TF, The cumulative volatilization of NH_3_ for seven consecutive days after topdressing nitrogen fertilizer supplement.

### Effect of Split Nitrogen Fertilizer Application on Soil Nitrogen Cycling

**FIGURE 2:**
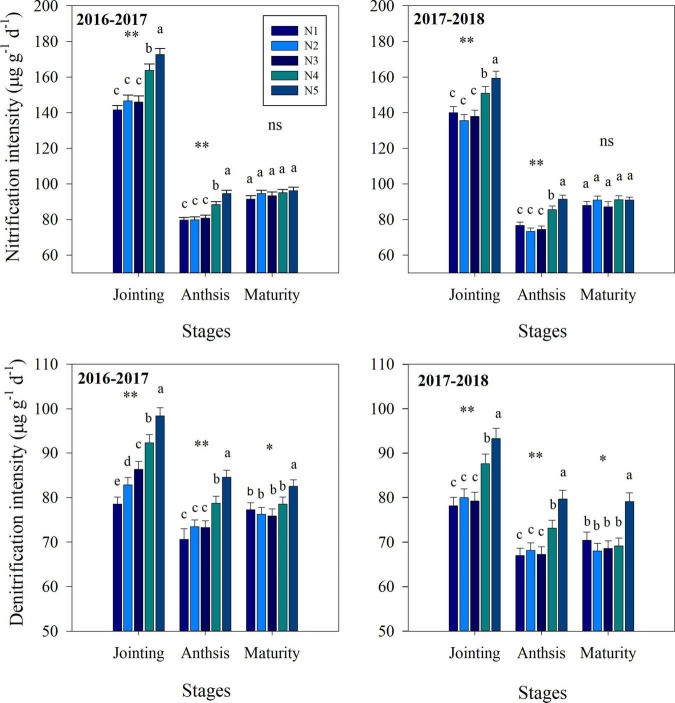
The effects of split nitrogen fertilizer on the nitrification intensity and denitrification intensity. *, ^**^ significant at the 0.05 and 0.01 probability levels, respectively, ns, no significant. Values followed by different letters within the same stages are significantly different at *P* < 0.05. N1 (basal/dressing, 100/0%), N2 (basal/dressing, 70/30%), N3 (basal/dressing, 50/50%), N4 (basal/dressing, 30/70%), and N5 (basal/dressing, 0/100%).

**FIGURE 3:**
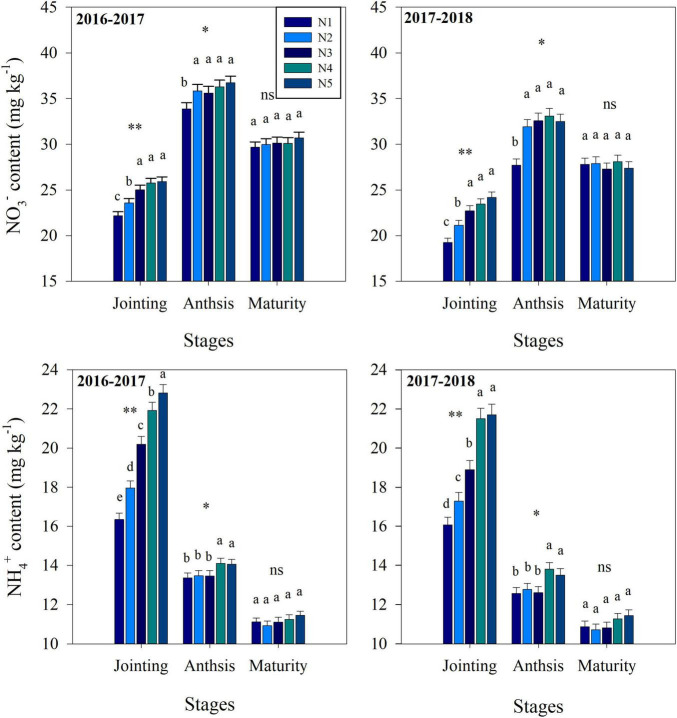
The effects of split nitrogen fertilizer on NO_3_^–^ content and NH_4_^+^ content. Different letters indicate significant differences between treatments (*P* < 0.05). *, ^**^ significant at the 0.05 and 0.01 probability levels, respectively, ns, no significant. N1 (basal/dressing, 100/0%), N2 (basal/dressing, 70/30%), N3 (basal/dressing, 50/50%), N4 (basal/dressing, 30/70%), and N5 (basal/dressing, 0/100%).

Fertilization strategy significantly affected soil nitrification, denitrification, NO_3_^–^ and NH_4_^+^ contents (Figures 2, 3). At the jointing stage, the nitrification and denitrification intensities increased with increasing topdressing nitrogen fertilizer proportion. Similarly, the soil NO_3_^–^ content and NH_4_^+^ content increased with increasing topdressing nitrogen fertilizer proportion. At the anthesis stage, the NH_4_^+^ content, nitrification and denitrification intensities under the N3 treatment were significantly lower than those under the N4 or N5 treatments, while the N1, N2, and N3 treatments did not differ significantly. However, the NO_3_^–^ content under the N3 treatment was significantly higher than that under N1 treatment, while the N2, N3, N4, and N5 treatments did not differ significantly. At the maturity stage, the split nitrogen treatments did not result in any significant differences in nitrification intensity, NO_3_^–^ content and NH_4_^+^ content. Additionally, the denitrification intensity under the N3 treatment was significantly lower than that under N5 treatment, while that under N1, N2, N3, and N4 treatments did not differ significantly.

### Effect of Split Nitrogen Fertilizer Application on Nitrogen Cycling Functional Genes

**FIGURE 4:**
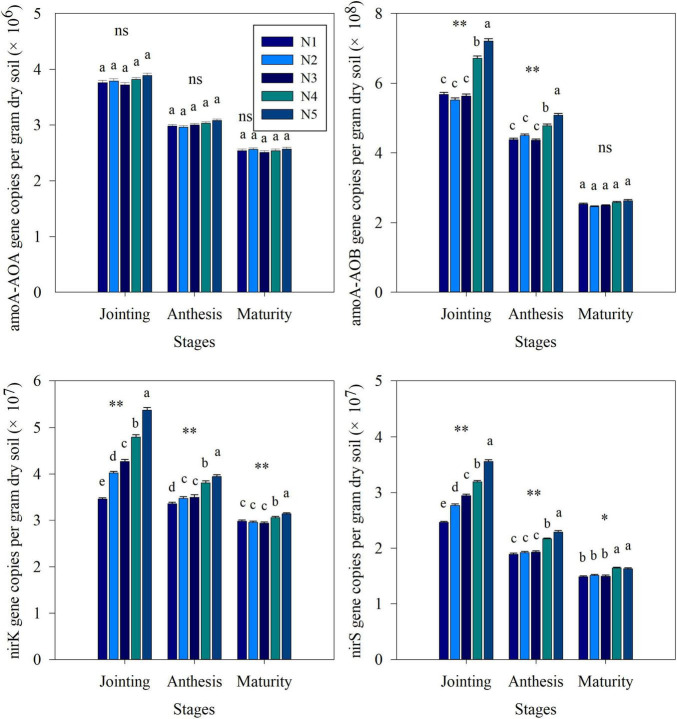
The effects of split nitrogen fertilizer on the abundance of *amoA-*AOA, ammonia monooxygenase gene in AOA populations; *amoA-*AOB, ammonia monooxygenase gene in AOB populations; *nirK*, Cu nitrate reductase gene; *nirS*, cd1 nitrate reductase gene. Different letters indicate significant differences between treatments (*P* < 0.05). *, ^**^ significant at the 0.05 and 0.01 probability levels, respectively, ns, no significant. N1 (basal/dressing, 100/0%), N2 (basal/dressing, 70/30%), N3 (basal/dressing, 50/50%), N4 (basal/dressing, 30/70%), and N5 (basal/dressing, 0/100%).

The q-PCR results were used to estimate the abundance of nitrogen cycle functional genes in soil under different split nitrogen fertilizer treatments (Figure 4). During the entire sampling process, changes in the *amoA-*AOA, *amoA-*AOB, *nirK*, and *nirS* counts in each group were similar, exhibiting gradual decreases. The fertilization strategies did not significantly affect the *amoA-*AOA counts. In all treatments, the *amoA-*AOB, *nirK*, and *nirS* counts at each stage increased with increasing proportion of topdressing nitrogen fertilizer at the jointing stage. During the anthesis and maturity stages, the *amoA-*AOB, *nirK*, and *nirS* counts were significantly lower under the N3 treatment than those under the N4 or N5 treatments, while the N1, N2, and N3 treatments did not differ significantly. Hence, a one-time addition of excess nitrogen fertilization can increase the abundance of key genes for nitrification and denitrification. Moreover, a reasonable ratio of basal to topdressing nitrogen fertilizer (N3) can ensure that the numbers of these genes remain at a low level.

### Wheat Nitrogen Utilization by ^15^N Tracer Technique

**FIGURE 5:**
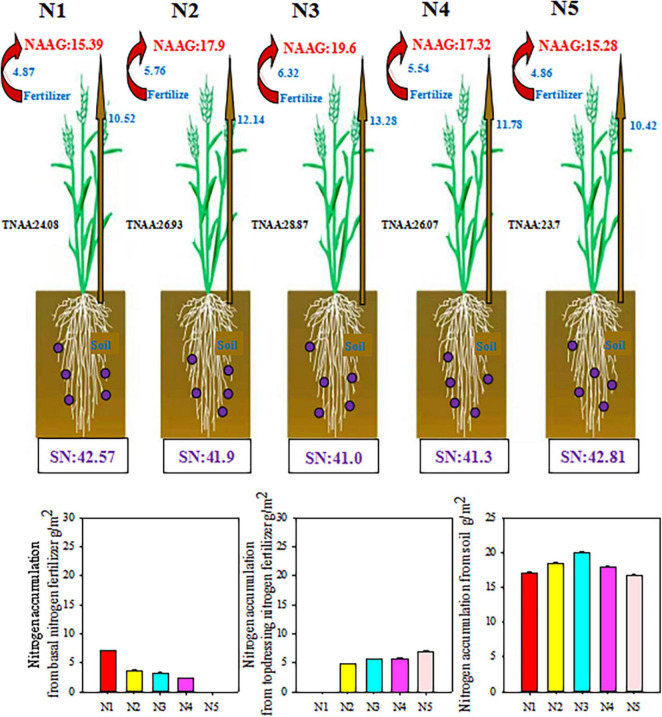
Effect of split nitrogen fertilizer on the plant nitrogen accumulation form fertilizer or soil. NAAG, nitrogen accumulation amount in grains; TNAA, Total plant nitrogen accumulation amount; SN, soil inorganic nitrogen content. N1 (basal/dressing, 100/0%), N2 (basal/dressing, 70/30%), N3 (basal/dressing, 50/50%), N4 (basal/dressing, 30/70%), and N5 (basal/dressing, 0/100%).

As shown in Figure 5, in addition to the nitrogen obtained from other crops that fix nitrogen, the nitrogen in winter wheat primarily originates from the soil and nitrogen fertilizer (Figure 5). Split nitrogen fertilizer treatment significantly affected nitrogen accumulation in wheat plant and grain. Generally, the plants treated with N3 absorbed the greatest amount of nitrogen from fertilizers and soil, followed by plants treated with N2 and N4, whereas those treated with N1 or N5 had the lowest levels. Moreover, the amount of nitrogen absorbed from the basal/topdressing nitrogen fertilizer increased with increasing basal/topdressing nitrogen ratio. Specifically, compared with those under N1, N2, N4, or N5 treatment, the total plant nitrogen accumulation amount (TNAA) under N3 treatment increased by 19.89, 7.20, 10.74, and 21.81%, respectively; while NAAG increased by 27.36, 9.50, 13.16, and 28.27%, respectively. These results demonstrate that N3 treatment was more conducive to nitrogen nutrient uptake by wheat plants, resulting in high-yield and high-efficiency.

**FIGURE 6:**
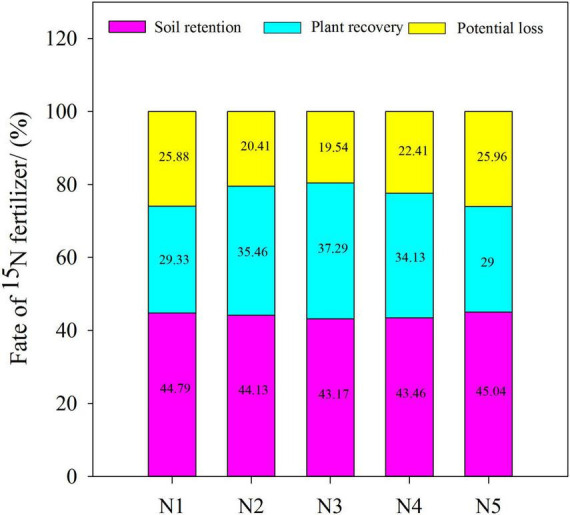
Effect of split nitrogen fertilizer on the fate of ^15^N fertilizer in plant-soil system. N1 (basal/dressing, 100/0%), N2 (basal/dressing, 70/30%), N3 (basal/dressing, 50/50%), N4 (basal/dressing, 30/70%), and N5 (basal/dressing, 0/100%).

We compared and analyzed the ratios of nitrogen fertilizer residue, plant recovery, and potential loss to nitrogen fertilizer application under the five treatments with split nitrogen (Figure 6). No significant difference was observed in the different ratios of nitrogen fertilizer residue to nitrogen fertilizer application. The ratio of plant recovery to nitrogen fertilizer application in N3 increased by 5.16–28.59% compared with those under N1, N2, N4, or N5 treatment. Similarly, under N3 treatment, the ratio of potential loss to nitrogen fertilizer application decreased by 12.81–24.73% compared with those under N1, N4, or N5 treatment. Increases in potential loss indicate that significant loss of nitrogen to the atmosphere occurs through other channels, resulting in environmental issues. Overall, N3 treatment significantly improved wheat plant nitrogen utilization, while decreasing nitrogen fertilizer loss.

## Discussion

### The Effect of Split Nitrogen Fertilizer on Soil Nitrogen Cycling

Traditional methods of applying nitrogen fertilizer in winter wheat planting are often inefficient (Nkebiwe et al., 2016), whereas split nitrogen fertilization may be a promising method to improve grain yield while reducing nitrogen loss (Afroz and Islam, 2014; Huda et al., 2016; Nkebiwe et al., 2016). Generally, NH_3_ volatilization has been an inevitable mode of nitrogen loss in agricultural fields, and is closely related to the nitrogen concentration and duration after fertilization (Yang X. D. et al., 2020). Our results show that application of nitrogen fertilization significantly influenced cumulative NH_3_ losses, which is consistent with the findings of Mkhabela et al. (2009). Cumulative NH_3_ loss from the different treatments accounted for 15.97–19.23% of the total nitrogen inputs over the 2 years. In contrast, it was evident that N3 treatments has the ability to reduce NH_3_ losses by 5.22–13.42% of the cumulative NH_3_ volatilization, with significant reductions of 9.20–21.36% and 9.91–22.78% in both the volatilization of NH_3_ and that of yield-scaled NH_3_, respectively, under the N3 treatment over the 2 years. It is evident that nitrogen loss *via* NH_3_ volatilization increases in conjunction with increasing proportions of basal or topdressing nitrogen (Li M. N. et al., 2018). Indeed, the current study was purposefully conducted in this region to demonstrate that NH_3_ volatilization from basal nitrogen fertilizer is higher than that from topdressing nitrogen fertilizer (Zhong et al., 2021). This may be due to immediate irrigation following topdressing nitrogen application causing infiltration of fertilizer into the deep soil, with subsequent reduction in the amount of ammoniacal nitrogen in the top soil layer. Additionally, wheat biomass is large at the jointing stage, thus, requiring a larger nitrogen supply. This would consequently lower the losses from the volatilization of NH_3_, which supports the findings from previous studies (Holcomb et al., 2011). In our study, the peak daily NH_3_ fluxes were identified 2–3 days after the application of nitrogen fertilizer, and they subsequently decreased to relatively low levels 6–7 days after application. These results suggest that the loss of NH_3_ primarily occurs during the early period following nitrogen application. In general, the duration of our gas sampling measurements following nitrogen fertilizer application could effectively capture most of NH_3_ volatilization induced by fertilizer application.

In addition, besides the large portion of nitrogen fertilizer losses being due to NH_3_ volatilization, the large amounts of unaccounted for nitrogen suggest that other loss pathways, such as nitrogen leaching, and soil nitrification/denitrification may also play a considerable role (Ju et al., 2009). Autotrophic and heterotrophic nitrification and denitrification are considered the primary sources of emissions by soil N_2_O (Cayuela et al., 2013; Wu et al., 2017). In our study, nitrogen fertilizer addition significantly alters the nitrification and denitrification intensity at the stages of wheat jointing, anthesis and maturity, suggesting that nitrogen fertilizer affects the current season’s wheat. The nitrification intensity at the heading is significantly and negatively correlated with nitrogen uptake by wheat, demonstrating that nitrification is a critical factor during the growth period of wheat (Yang et al., 2017). Moreover, *amoA-*AOA and *amoA-*AOB are two critical groups that participate in nitrification. The findings of our study indicate that nitrification activity was stimulated by large topdressing nitrogen fertilizer proportions and was accompanied by a significant increase in the abundance of AOB. AOB can frequently outcompete AOA for inorganic nitrogen fertilizer (Hink et al., 2017). This competition can include the inhibition of AOA functions and growth, which prefer to use native soil nitrogen in contrast to an exogenous nitrogen source as a substrate (Fisk et al., 2015). The increase in soil nitrification could result in loss of nitrogen from agricultural systems and subsequent pollution of groundwater owing to nitrate leaching and denitrification. Our results also show that larger topdressing nitrogen fertilizer proportions produce a higher average accumulation of nitrate and ammonium in soil than other split nitrogen fertilizer ratios.

Previous research has shown that total N_2_O emissions positively correlated with soil denitrification intensity (Fan et al., 2018), which is closely related to the size of pool labile nitrogen forms, such as NH_4_^+^, NO_3_^–^. In this study, compared with N4 and N5 treatments, N3 treatment resulted in a significant decrease in the concentrations of NH_4_^+^ and NO_3_^–^, suggesting that this split nitrogen fertilizer ratio reduces urea degradation and that such treatment improves the absorption of NH_4_^+^, NO_3_^–^ (Lu et al., 2014; Liu et al., 2017; Li Y. F. et al., 2018). This relationship was confirmed by Harter et al. (2014), who found that the N_2_O emissions from the soil are indirectly reduced by a decrease in the concentrations of NH_4_^+^ and NO_3_^–^. Moreover, split nitrogen fertilizer can alter the rates of soil denitrification by altering the microbial and chemical properties of the soil (Nguyen et al., 2017; Li Y. F. et al., 2018). For example, managing nitrogen fertilizer appropriately can reduce the gross rates of soil denitrification by altering the community structure of soil AOB (Dempster et al., 2012; Li Y. F. et al., 2018). Weedon et al. (2012) also reported that *nirK* and *nirS* serve as the predominant genes in soil denitrification, the abundance of which was significantly reduced in our study following application of an optimal split nitrogen fertilizer ratio compared with N4 and N5 treatments. A possible explanation for this observation could be that compared with other treatments, N3 treatment maintains the low contents of NH_4_^+^ and NO_3_^–^ in the soil at anthesis and maturity, and thus, denitrifying bacteria only exhibit low activity levels. Along with the increased soil denitrification rates following split nitrogen fertilizer application, these results suggest that N3 treatment decreases soil N_2_O emission by reducing nitrification and denitrification rates. Based on these data, we conclude that the optimal split nitrogen fertilizer application ratio decreases soil nitrogen losses by decreasing the concentration of labile nitrogen, and the abundance of nitrogen cycling key genes, as well as the rates of nitrification and denitrification in wheat-land.

### The Effect of Split Nitrogen Fertilizer on Wheat Nitrogen Utilization by ^15^N Tracer Technique

In the ^15^N tracer experiment, separate applications of basal fertilizer ^15^N and topdressing fertilizer ^15^N were used to overcome issues associated with utilization of traditional fertilizers, which only examine the utilization of total nitrogen fertilizer during the process of wheat growing. Based on measurements in soil samples and wheat plants, we estimated the applications of basal and topdressing nitrogen fertilizers in the wheat-soil system and the accumulation of nitrogen from basal/topdressing fertilizer in the system of wheat and soil. The results indicated that the N3 treatment resulted in a higher TNAA of wheat to fertilizer ^15^N than that under other split nitrogen fertilizer treatments. The TNAA in wheat was increased by 7.20–21.81% relative to that from fertilizer ^15^N, and NAAG (9.50–28.27%) was the highest under N3 treatment. In addition, under N3 treatment, the nitrogen accumulation from soil increased by 7.20–27.45%, compared with that under other split nitrogen fertilizer treatments. These results indicate that the N3 treatment contributes to a high accumulation of nitrogen by improving the absorption and utilization of soil and fertilizer nitrogen by wheat (Shi et al., 2012). In fact, a single application of excess fertilization resulted in a soil nitrogen surplus due to a net difference in the nitrogen supply and demand of the crop (Fageria and Baligar, 2005).

Previous studies have shown that NO_3_^–^ leaching causes excessive loss of nitrogen and pollute the environment (Oborn et al., 2003; Sieling and Kage, 2006). Our results indicate that the plants accumulate higher amounts of nitrogen when treated with topdressing, rather than basal fertilizer. Thus, applications of basal nitrogen and high-level topdressing with nitrogen lead to a surplus of soil nitrogen and possibly loss *via* NO_3_^–^ leaching. Loss of basal nitrogen results in a loss of nitrogen throughout the entire growing season due to the poor synchrony between the supply of nitrogen and the demand of crops. Further evidence suggests that altering the type of nitrogen fertilizer and applying it at the optimal rates for fertilization can meet the dual goals of sustaining the accumulation of nitrogen in crops and mitigating the volatilization of NH_3_ and greenhouse gases in winter wheat systems.

Our study confirms that nitrogen fertilizer has high potential for NH_3_ loss during winter wheat production in NCP. Considering that the modes of split nitrogen fertilizer in the present study effectively decrease NH_3_ losses from wheat fields, while significantly improving nitrogen use efficiency, local farmers would be encouraged to adopt this split nitrogen fertilizer technology. Nitrogen fertilizer management has also been reported to regulate the amount of plant available nitrogen by influencing the soil biochemical process-nitrogen transformation dynamics; thus, they affect plant nitrogen use efficiency (Zhao et al., 2018). More importantly, further direct evidence between nitrogen loss and specific nitrogen transformation process need to be established. As mentioned previously, crucial evidence has indicated that more microbial community structure was observed (Reichel et al., 2018). Thus, further study is needed to understand the unique behavior of nitrogen fertilizer practices on nitrogen transformation rates and the microbial community structure associated with soil nitrogen cycling.

## Conclusion

Our results highlighted the effects of split nitrogen fertilization on improving wheat nitrogen use efficiency, as well as the underlying mechanism associated with decreasing nitrogen loss. In this study, compared with other treatments, N3 treatment significantly decreased soil NH_3_ volatilization, resulting in a lower overall environmental burden. Meanwhile, the N3 treatment improved nitrogen use efficiency mainly due to increasing nitrogen accumulation in grans in the winter wheat cropping system. Appropriately splitting nitrogen fertilizer applications may reduce the nitrification and denitrification activities by decreasing the substrate (NO_3_^–^, NH_4_^+^) concentration, reducing *amoA-*AOB, *nirS*, and *nirK* gene abundance, while also more closely matching wheat nitrogen uptake and reducing nitrogen fertilizer loss. Thus, appropriately splitting nitrogen fertilizer applications under water-saving irrigation conditions is an effective fertilization strategy with benefits for both agronomy and the environment. Gaseous loss of nitrogen fertilizer, when nitrogen fertilizer is surface-applied, is the main nitrogen loss pathway in agroecosystems and is strictly tied to soil microbial activities. Future studies on nitrogen fertilizer management effects should focus on the direction of the urea-derived nitrogen loss and soil microbial community structure.

## Data Availability Statement

The original contributions presented in the study are included in the article/Supplementary Material, further inquiries can be directed to the corresponding author.

## Author Contributions

ZZ: data curation, formal analyses, and writing original draft. YZ and ZY: funding acquisition. ZZ and YS: investigation. YZ: project administration. All authors contributed to the writing review and editing and read and agreed to the published version of the manuscript.

## Conflict of Interest

The authors declare that the research was conducted in the absence of any commercial or financial relationships that could be construed as a potential conflict of interest.

## Publisher’s Note

All claims expressed in this article are solely those of the authors and do not necessarily represent those of their affiliated organizations, or those of the publisher, the editors and the reviewers. Any product that may be evaluated in this article, or claim that may be made by its manufacturer, is not guaranteed or endorsed by the publisher.
