# Supplementary material for: Impacts of Fertilization Optimization on Soil Nitrogen Cycling and Wheat Nitrogen Utilization Under Water-Saving Irrigation
Source: Front Plant Sci. 2022 May 19;13:878424. doi: 10.3389/fpls.2022.878424 (PMC9161168; doi:10.3389/fpls.2022.878424)
Supplement: Supplementary file 1 [file Data_Sheet_1.doc]

Table S1 | Greenhouse gas and soil sampling dates for the different growth stages during the 2016-2017 and 2017-2018 growing seasons.

| Stage | SO | TI | OV | TG | JT | BT | AT | 7d | 14d | 21d | 28d | MA |
| --- | --- | --- | --- | --- | --- | --- | --- | --- | --- | --- | --- | --- |
| Time（2016-2017） | 10.12 | 11.03 | 11.29 | 03.03 | 04.06 | 04.20 | 04.28 | 05.06 | 05.13 | 05.20 | 05.27 | 06.09 |
| Time（2017-2018） | 10.24 | 11.18 | 12.11 | 03.08 | 04.03 | 04.16 | 04.29 | 05.07 | 05.14 | 05.21 | 05.28 | 06.07 |

SO, sowing; TI, tillering; OV, overwintering; TG, turning green; JT, jointing; BT, booting; AT, anthesis; GF, grain-filling; MA, maturity.

Table S2 | Primers used for quantitative PCR.

| Target  group | Primer | Sequence(5-3) | Length of amplicon | Thermal profile | Reference |
| --- | --- | --- | --- | --- | --- |
| *amoA*-AOA | Arch-amoA26F/Arch-amoA417R | GACTACATMTTCTAYACWGAYTGGGC/GGKGTCATRTATGGWGGYAAYGTTGG | 415 | 5 min at 95 ℃ followed by 40 cycles of 15 s at 95 ℃ and 30 s at 60 ℃ | (Park et al., 2008) |
| *amoA*-AOB | amoA-F/amoA-R | GGGGTTTCTACTGGTGGT/CCCCTCKGSAAAGCCTTCTTC | 491 | 5 min at 95 ℃ followed by 40 cycles of 15 s at 95 ℃ and 30 s at 60 ℃ | (Rotthauwe et al., 1997) |
| *nirK* | nirK-1479yF/  nirK-HIR | ATCGGCGGYRAAGGCGA/GCCTCGATCAGRTTRTGGTT | 164 | 95 ℃ for 5 min, 40 cycles at 95 ℃ for 5 s, 62 ℃ for 30 s, 95 ℃ for 10 s (+0.3) | (Usyskin-Tonne et al., 2020) |
| *nirS* | nirS128 43F/nirS128 1665R | CTGCTCGGTCTGGCAGTT/TGGGGTGATCAACCTTTTGT | 1622 | 95 ℃ for 5 min, 40 cycles at 95 ℃ for 5 s, 62 ℃ for 30 s, 95 ℃ for 10 s (+0.3) | (Usyskin-Tonne et al., 2020) |


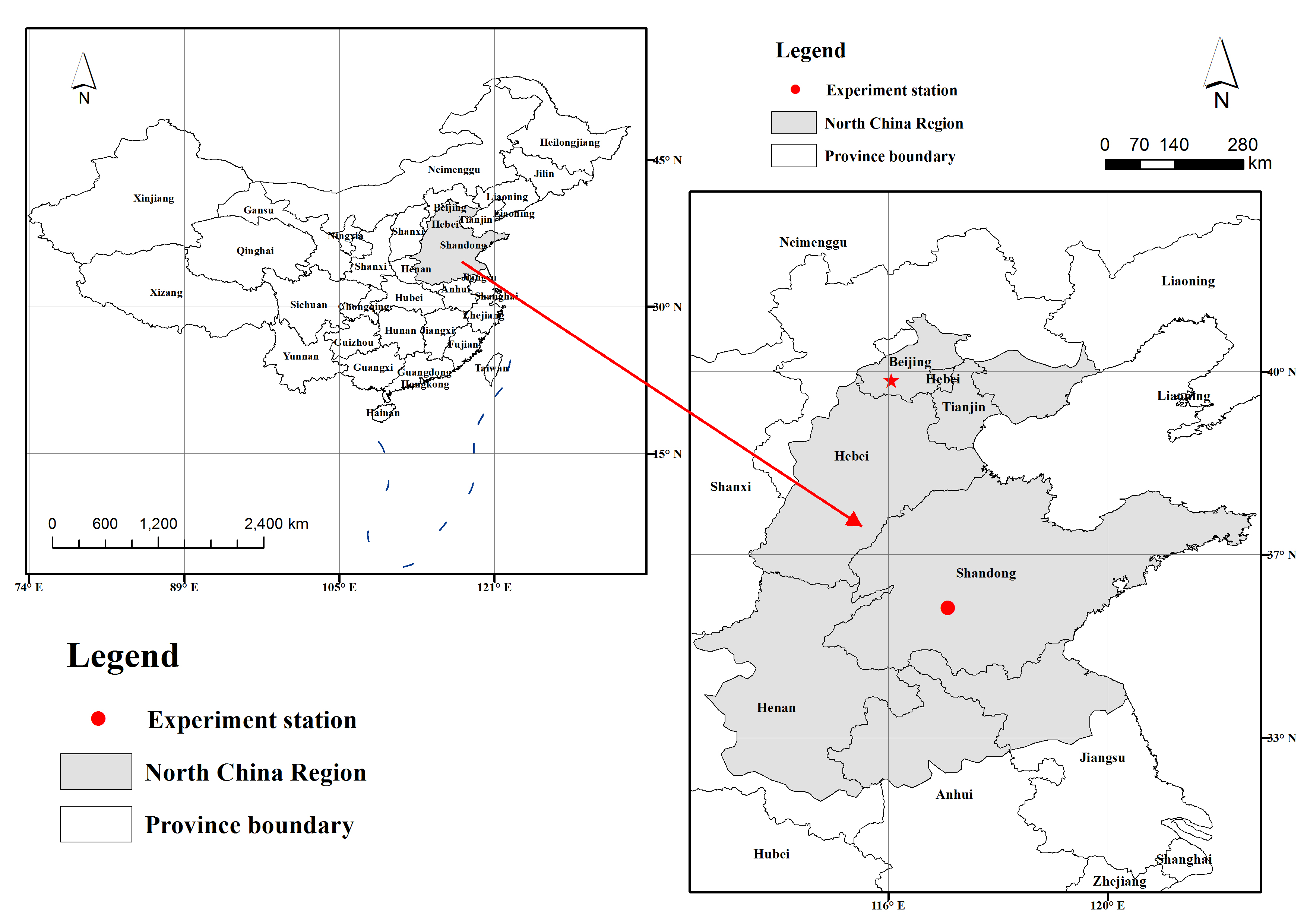


FIGURE S1 | Map showing the study site.

FIGURE S2 | Effective precipitation during wheat growth period.


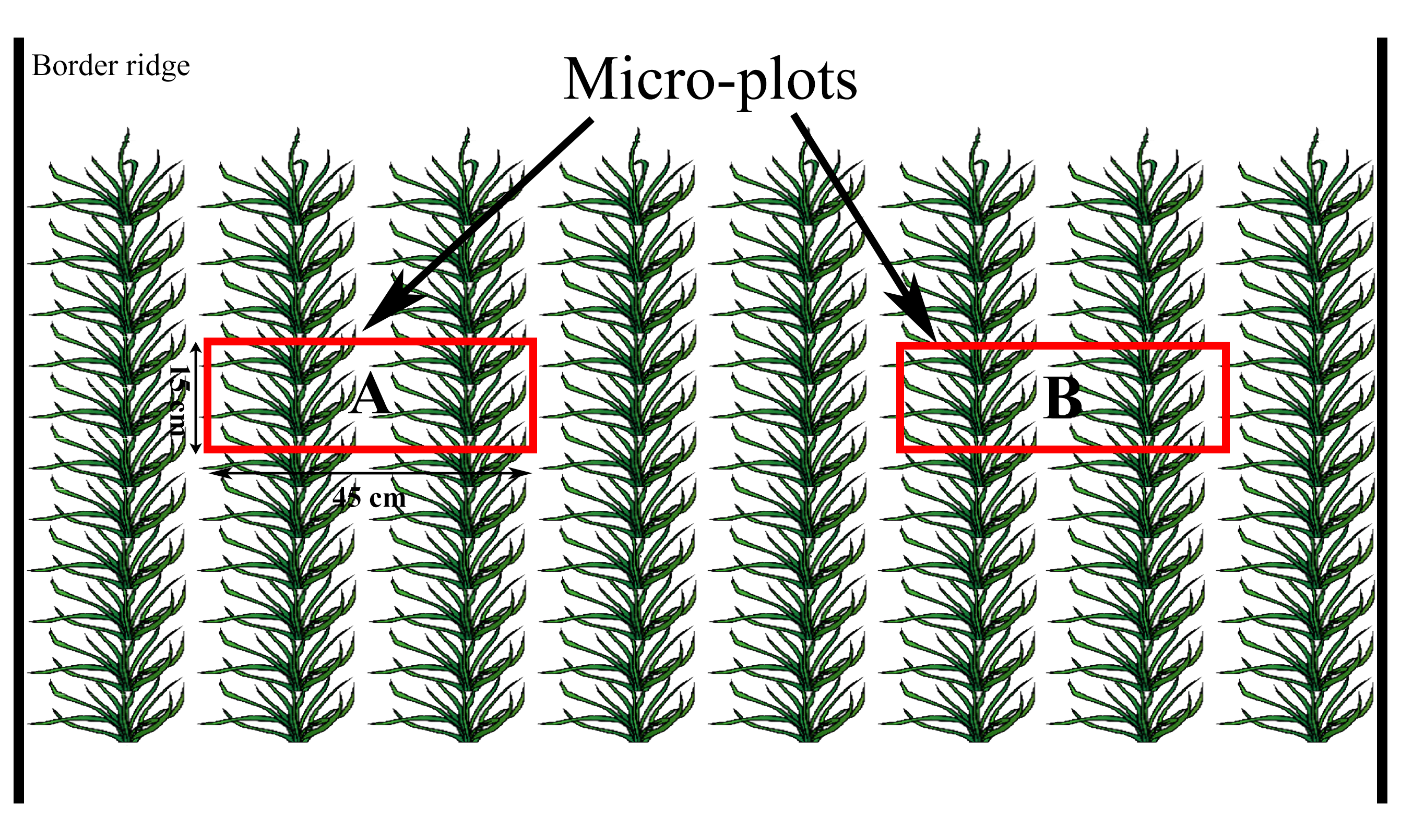


FIGURE S3 | Map showing the micro-plots.

**References**

Park, S. J., Park, B. J., Rhee, S. K., 2008. Comparative analysis of archaeal 16S rRNA and amoA genes to estimate the abundance and diversity of ammonia-oxidizing archaea in marine sediments. Extremophiles. 12, 605-615. doi:10.1007/s00792-008-0165-7.

Rotthauwe, J. H., Witzel, K. P., Liesack, W., 1997. The ammonia monooxygenase structural gene *amoA* as a functional marker, molecular fine-scale analysis of natural ammonia-oxidizing populations. Appl Environ Microb. 63, 4704-4712. doi:10.1126/science.284.5411.63.

Usyskin-Tonne, A., Hadar, Y., Yermiyahu, U., Minz, D., 2020. Elevated CO_2_ has a significant impact on denitrifying bacterial community in wheat roots. Soil Biol. Biochem. 142, 107697. doi:10.1016/j.soilbio.2019.107697.
